# Panicle blast 1 (Pb1) resistance is dependent on at least four QTLs in the rice genome

**DOI:** 10.1186/s12284-017-0175-0

**Published:** 2017-08-01

**Authors:** Haruhiko Inoue, Mitsuru Nakamura, Tatsumi Mizubayashi, Akira Takahashi, Shoji Sugano, Shuuichi Fukuoka, Nagao Hayashi

**Affiliations:** 1 NARO Institute of Agrobiological Sciences (NIAS), 2-1-2 Kan-nondai, Tsukuba, Ibaraki, 305-8602 Japan; 2 Mountainous Region Agricultural Research Institute, Aichi Agricultural Research Center, Inabu, Toyota, Aichi 441-2513 Japan; 3 NARO Institute of Crop Science, Kannondai 2-1-18, Tsukuba, Ibaraki, 305-8518 Japan

**Keywords:** *Oryza sativa*, *Pyricularia oryzae*, Panicle blast, QTL, Salicylic acid, Resistance gene

## Abstract

**Background:**

Rice blast is the most serious disease afflicting rice and there is an urgent need for the use of disease resistance (R) genes in blast tolerance breeding programs. *Pb1* is classified as a quantitative resistance gene and it does not have fungal specificity. *Pb1*-mediated resistance develops in the latter stages of growth. However, some cultivars, such as Kanto209 (K209), cultivar name Satojiman, despite possessing *Pb1*, do not exert resistance to rice blast during the reproductive stage.

**Results:**

We found that the expression of *WRKY45* gene downstream of *Pb1* was weakly induced by rice blast inoculation at the full heading stage in K209. Genetic analysis using the SNP-based Golden Gate assay of K209 crossing with Koshihikari Aichi SBL (KASBL) found at least four regions related to the resistance in the rice genome (Chr8, Chr9, Chr7, Chr11). Mapping of QTL related to Chr7 confirmed the existence of factors that were required for the resistance of *Pb1* in the 22 to 23 Mbp region of the rice genome.

**Conclusion:**

We clarified how the K209 cultivar is vulnerable to the blast disease despite possessing *Pb1* and found the DNA marker responsible for the quantitative resistance of *Pb1*. We identified the QTL loci required for *Pb1*-mediated resistance to rice panicle blast. *Pb1* was negatively dependent on at least three QTLs, 7, 9 and 11, and positively dependent on one, QTL 8, in the K209 genome. This finding paves the way for creating a line to select optimal QTLs in order to make use of *Pb1*-mediated resistance more effectively.

**Electronic supplementary material:**

The online version of this article (doi:10.1186/s12284-017-0175-0) contains supplementary material, which is available to authorized users.

## Background

Rice blast is a pathogenicity that affects rice plants worldwide. In order to minimize the damage of rice blast, resistance (R)-gene cultivars are commonly used. The use of R genes to develop resistant cultivars has become an urgent goal in rice breeding programs. Several molecular approaches to study rice blast resistance genes have been developed during the last decade. So far, hundreds of blast *R* genes have been mapped in the rice genome. A large group of *R* genes is clustered in several genomic regions reviewed in (Liu et al. 2014). Most of the genes encode nucleotide-binding site (NBS) leucine-rich repeat (LRR) proteins that interact with pathogen effectors and trigger defense reactions following a gene-for-gene model of recognition (Bryan et al. 2000; Okuyama et al. 2011; Cesari et al. 2013). One of the plant defense mechanisms is the recognition of Microbe-Associated Molecular Patterns (MAMPs) by receptors, leading to generally weak defense responses called MAMP-Triggered Immunity (MTI). Some pathogens overcome the resistance by secreting effector proteins that interfere with host resistance and promote pathogenicity. Plants have evolved to recognize against the effectors through Effector-Triggered Immunity (ETI). True resistance genes are specific and provide strong resistance to the rice blast, but the effect of R genes decline after 2 or 3 years.

In contrast to MTI or ETI, the quantitative resistance genes are durable to the blast, but are less understood (Cook et al. 2015; Fukuoka et al. 2015). Recently, intensive genetic analyses of quantitative “leaf” blast resistance genes including *pi21*, *Pi34*, *Pi35*, *Pi39 and Pi63* have been carried out (Nguyen et al. 2006; Zenbayashi-Sawata et al. 2007; Terashima et al. 2008; Fukuoka et al. 2009; Xu et al. 2014). In contrast to these leaf blast resistance genes, the panicle blast (*Pb1*) resistance gene has been used as the durable form that confers rice plants partial resistance to the rice blast with no fungal race specificity (Fujii et al. 2000). *Pb1* gene has been introduced into rice cultivars in Korea (Lee et al. 2015). The cultivars have remained resistant to panicle blast for over 35 years. Cloning of *Pb1* has identified that the resistance is weak on the early stage and gradually becomes strong for the reproductive stage of the plant, and is dependent on the *Pb1* expression (Hayashi et al. 2010). After heading stage, the resistance of *Pb1* cultivar is very strong to rice blast.

The resistance mechanism of *Pb1* is attributed to its interaction with WRKY45 (Inoue et al. 2013), which plays a crucial role in the salicylic acid (SA) pathway in rice immunity (Shimono et al. 2007). WRKY45 is regulated by a ubiquitin-proteasome system in the rice nucleus (Matsushita et al. 2012). The Pb1 coiled-coil domain interacts with the nuclear-localized WRKY45 (Inoue et al. 2013), resulting in a Pb1-WKRY45 complex is a weaker target than WRKY45 for protein degradation. WRKY45 overexpressed transgenic rice plants have strong resistance not only to the leaf rice blast but also to the panicle blast (SHIMONO et al. 2011). The genes in the downstream pathway of WRKY45, including *OsNAC4*, *OsHSF1*, *OsOPR4* (Nakayama et al. 2013), and diterpenoid phytoalexin (DP), a bHLH transcription factor (Yamamura et al. 2015), play a crucial role in disease resistance. Cytokinins (CKs) play a role in mediating the signal of *Pyricularia oryzae* infection to trigger the induction of DP biosynthetic genes in benzothiadiazole- (BTH) primed plants (AKAGI et al. 2014).

Kanto 209 (K209) cultivar is one of the cultivars harboring *Pb1*; however, the resistance of the cultivar is weak in comparison with other *Pb1* cultivars. In the K209 cultivar of rice, the expression of WRKY45 is lower than that of Koshihikari Aichi SBL (KASBL). The lower expression of the gene might be due to the genotype of the cultivar. To identify the mechanism of this rice blast resistance, we crossed the K209 and KASBL cultivars. Genomic DNA of their F_3_progeny was subjected to SNP-based Golden Gate assay and we found at least four quantitative trail loci (QTL) to the panicle rice blast resistance. Among the four QTLs, we focused on QTL7, which was located on chromosome 7 and showed *Pb1*-dependent defense against panicle blast in field tests, and performed a map-based cloning.

## Results

**Fig. 1:**
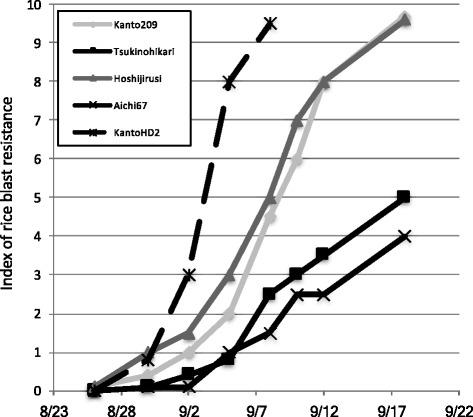
*Development of panicle blast on Pb1*-mediated *resistance gene cultivars*. Rice blast index of cultivars grown in an experimental paddy field at the Aichi Agricultural Research Center field in 2014. The cultivars, Aichi67, Tsukinohikari, Hoshijirushi, K209 and Kanto HD2 were evaluated in the field. The resistances of Tsukinohikari and Aichi 67 were stronger than that of Kanto 209 and Hoshijirushi in the full heading stage. The heading date of Aichi67, Tsukinohikari, Hoshijirushi, K209 and Kanto HD2 were 21st, 20th, 20th, 19th and 22nd of August, 2013, respectively. The Kanto HD2 cultivar was the negative control for the rice panicle blast resistance

Field tests were performed to examine the resistance to rice blast on a large number of *Pb1* cultivars available in Japan. To test the resistance, we evaluated the index of panicle blast severity of the cultivars at the Aichi Agricultural Research Center, Mountainous Resin Agricultural Research Institute. A lower index is indicative of higher resistance to panicle blast. Figure 1 shows the gradual increase in severity index to panicle blast with time, and it is due to the high pressure of the rice blast. The resistance of Tsukinohikari and its sister cultivar Aichi67 were stronger than that of K209 and its progeny Hosijirushi (Fig. 1). These cultivars had similar full heading date within plus or minus 2 days from August 20th 2014. The resistance of Kanto HD2, which does not harbor *Pb1*, was similar to the other cultivars close to the heading date (Fig. 1).

### K209 has other potential mutations for blast resistance

**Fig. 2:**
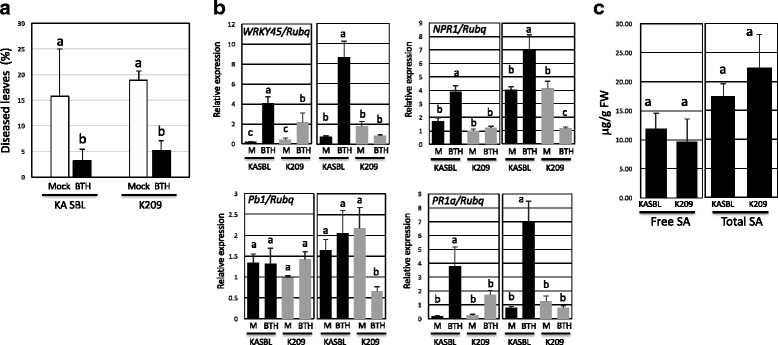
BTH-induced rice blast resistance of 5th leaves, expression analysis by q-RT-PCR, and the concentration of SA in KASBL and K209 cultivars. **a**. The 5th leaves of KASBL and K209 were sprayed with 50 μΜ BTH or water. After 2 days, the leaves were spray-inoculated with blast fungal conidia suspension (isolate Kyu89–246, 4.0 × 10^4^ spores ml^−1^). Disease symptoms were evaluated by percentage of diseased leaves 10 days after inoculation. The experiments were performed three times with similar results. **b**. The expression of *Pb1*, *WRKY45, NH1* and *PR1a* in the 5th leaves were analyzed by q-RT-PCR. Leaves after mock (M) or BTH treatment (BTH) for 2 days (left column) or 4 days (right column) were used for the expression analyses. The Y-axis indicates the mRNA expression relative to that of UBQ. The averages with SD are shown. Different letters indicate significant differences among the different lines at *p* < 0.05 (one-way ANOVA). Experiments were repeated twice with similar results. **c**. Free (*left*) and total (*right*) SA content of 25th leaves of KASBL and K209 measured by high performance liquid chromatography (HPLC). Letters indicate significant differences among the different lines at *p* < 0.05 (one-way ANOVA). Experiments were repeated three times with similar results

The only information available on *Pb1*-mediated resistance is that it is dependent on *WRKY45* (Inoue et al. 2013), so we checked the sequence of *Pb1* and *WRKY45* in the K209 cultivar at the DNA and RNA level. At the DNA level, K209 and KASBL were sequenced for the *Pb1* and *WRKY45* regions. DNA from plants was extracted using CTAB method and the *Pb1* and *WRKY45* regions were amplified using PCR. The sequences of PCR products were then determined using specific primers on an ABI sequencer. As a result, there were no sequence differences in the *Pb1* open reading frame (ORF) or the 2 kb upstream sequence between the KASBL and K209 cultivars (data not shown). Similarly, no differences were observed in the sequence of *WRKY45*, including the promoter region of 1.7 kbp with ORF and 3′ region site (data not shown). Previously, the expression of WRKY45 in rice was shown to be markedly enhanced by BTH treatment and resulted in resistance to rice blast fungus, whereas RNA interference–mediated knockdown of WRKY45 compromised BTH-inducible resistance to blast disease (Shimono et al. 2007). So we tested if the WRKY45 sequence still remains intact in the K209 cultivars and found that K209 cultivar treated with BTH exhibited resistance for rice blast while nontreated plants showed susceptibility (Fig. 2a).

To study gene expression by quantitative PCR, total RNAs were extracted from the 25 days old leaf of KASBL and K209 after treatment with water (mock) or BTH for 2 or 4 days. Surprisingly, *WRKY45*, *NH1* and *PR1a* expression was reduced by BTH treatment on both days (Fig. 2b). The transcript abundance for *Pb1* was significantly reduced at 4 days after treatment with BTH (Fig. 2b). The concentrations of SA and total SA were not statistically different between K209 and KASBL plants (Fig. 2c).

**Fig. 3:**
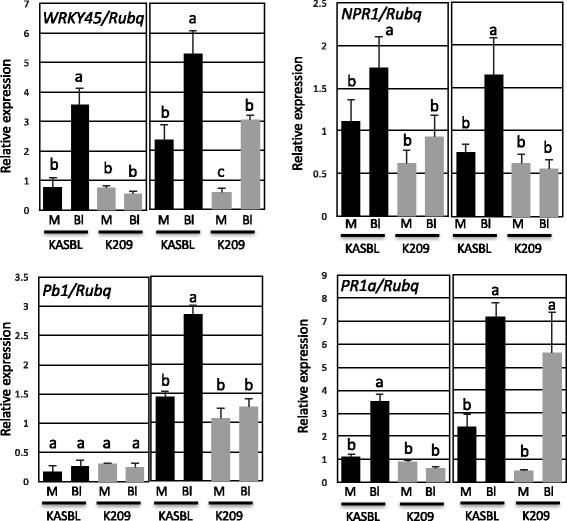
Expression analyses in flag leaves in response to rice blast. The flag leaves of KASBL and K209 were spray-inoculated with water (mock) or blast fungal conidia suspension (Bl) (2.0–1.5 × 10^5^ spores ml^−1^). The expression of *NH1*, *WRKY45 Pb1* and *PR1a* in flag leaves 1 day (left column) and 3 days (right column) after inoculation was analyzed by q-RT-PCR. The Y-axis shows relative mRNA expression to UBQ. The average of 12 plants and SD are shown. Different letters indicate significant differences among the different lines at *p* < 0.05 (one-way ANOVA). Experiments were repeated twice with similar results

To study gene expression, RNA was extracted from K209 and KASBL during the reproductive growth phase. The flag leaves with or without induced rice blast after 1 and 3 days were removed from K209 and KASBL cultivars, RNAs were extracted and quantitative PCR was performed. The *Pb1* transcript of K209 exhibited reduced expression compared to KASBL after inoculation (Fig. 3). The WRKY45 transcript of KASBL was induced 1 day and 3 days after the rice blast (Fig. 3). The transcript abundance of *WRKY45* was reduced and the expression of *NH1* in K209 still remained at the basal level after rice blast inoculation (Fig. 3), suggesting that the transport or the perception of SA was defective. These indicates that the mutation(s) was (were) not in the *Pb1* genomic region in the genome of K209.

### K209 has at least four DNA mutations for blast resistance on its genome

**Fig. 4:**
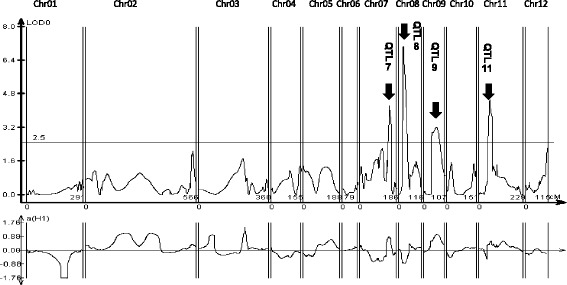
LOD curve plot from QTL analysis for panicle blast resistance tested by using 82 F_4_ lines from the K209 and KASBL cross evaluated in 2010. Horizontal line at 2.5 indicates LOD thresholds obtained by using 1000 permutations

It was apparent that K209 had a genomic alteration in the molecular mechanism responsible for *Pb1*-mediated resistance gene activation. To identify this mechanism, K209 line (+*Pb1*) was crossed with KASBL (+*Pb1*). Genomic DNA was extracted from 82 F_3_ progeny from each line and subjected to the SNP-based Golden Gate assay. Out of 768 SNPs spanning the entire genome, 199 SNPs distinguished K209 from KASBL. The ratio of heterozygosity in the samples was 26.9%. To evaluate rice panicle blast resistance, the F_4_ plants were tested in an experimental field at the Aichi Agricultural Research Center in 2010. QTL analysis detected four QTLs for *Pb1*-mediated resistance each one on chromosomes 7, 8, 9 and 11 (Table 1). The alleles from K209 at the QTL 7, 9 and 11 had negative effect on rice blast resistance while that on QTL 8 had positive effect (Fig. 4). QTL7 was located on the long arm, the end of chromosome 7 while QTL9 was on the middle of the long arm. QTL11 and QLT8 were on the short arm, with the former present near the centromere and the latter near the telomere. The effects of resistance QTL alleles of K209 and KASBL on panicle blast resistance to different haplotypes are shown in Table 2.

**Table 1:** Putative QTLs associated with panicle blast resistance in K209/KASBL F_4_ lines in 2010

Nearest markers	chromosome	Logarithm of odds	Additive effect^a^	Phenotype variation^b^ explained (%)
b297 = C7–14	7	4.24	0.74	12.1
b063 = C8–4	8	7.06	−0.94	22.0
b586 = C9–7	9	3.30	0.86	14.6
b285 = C8–4	11	4.52	0.58	12.0

^a^Additive effects of K209 allele

^b^Phenotypic variation explained by QTL

**Table 2:** Eight genotypes defined by four QTLs for panicle blast resistance and their phenotypes

Lines	QTLs				Leaf disease index	Panicle disease index	Days until heading
	QTL7	QTL8	QTL9	QTL11			
Ha21	+	+	+	+	3	1.8	76
Ha55	+	+	+	+	5	2.8	81
Ha06	+	+		+	5	2.6	73
Ha64	+	+	+		5.5	2.8	76
Ha13	+	+		+	6	3.6	76
Ha30	+	+		+	6	4.1	76
Ha38		+	+	+	6.5	4.5	83
Ha27	+		+	+	5	5.0	65
Ha12		+			6	5.6	76
Ha22					5	7.2	73
Ha29					5	8.8	70
K209		+			5	7.6	80
KASBL	+		+	+	5	4.0	70

+: homozygous resistance QTL allele

### QTL7 was located within a 1.8 Mbp region on chromosome 7

**Fig. 5:**
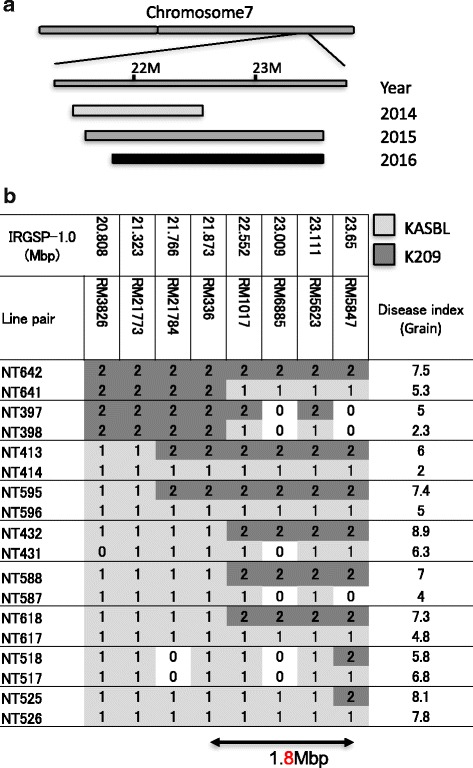
**a** QTL region for panicle blast resistance on chromosome 7 estimated by a 3 year field test using recombinant inbred lines with recombination within and around the QTL. **b** Graphical genotypes of recombinant inbred lines used to determine the QTL7 locus. Positions are based on the International Rice Genome Sequencing Project 1.0 of the Nipponbare genome. Box 1 indicates chromosomes derived from the resistance cultivar, KASBL; box 2, from the susceptible cultivar K209. Box 0 indicates missing data. The location of the QTL7 locus, indicated at the bottom, is based on phenotypic data obtained from field tests obtained in 2016

To delimit the chromosomal region for QTL7 through a map-based procedure, we screened recombinants within and around this QTL in a mapping population and subjected them to a field test. We compared the degree of rice blast resistance between recombinants and their counterpart non-recombinant controls. We evaluated 14 lines in 2014, 122 lines in 2015, and 86 lines in 2016. The positions of QTLs identified by the 3-year field test are shown in Fig. 5a. When disease index differs by 2 or more, we judged that the resistance of two lines is different. We found that NT642/641, NT397/398, NT413/414, NT595/596, NT432/431, NT588/587 and NT618/617 exhibited differences in the panicle rice blast. Two genotypes, NT518/517 and NT525/526 showed no difference in panicle resistance index (Fig 5b). The results led to the conclusion that QTL7 lies in the 1.777 Mb region within the marker loci between *RM336* and *RM5847* (Fig. 5b).

## Discussion

### *Rice with enhanced Pb1*-mediated *resistance can be produced by using QTL Analyses*

*Pb1* is known as a durable blast-resistance gene consisting of a single dominant locus. Introduction of *Pb1* gene into rice plants by marker selection breeding has been actively carried out. As a result, there are several *Pb1* cultivars to panicle blast resistance of rice in Japan. Among them, K209 and its progeny cultivar Hoshijirushi have been found to exhibit weak resistance to rice panicle blast despite harboring *Pb1*. The loss of *Pb1*-mediated resistance over time has generated a negative impact for rice breeders for a long time. To this end, we attempted to identify the mechanism of the loss of *Pb1*-mediated blast resistance of *Pb1* in K209. Traditionally, a functional gene region is introduced into a genomic fragment, so the gene can have a large additive effect in breeding. However, interactions with the genes in the background have been known to cause differential effects with respect to rice bacterial blight resistance (Sun et al. 2004; Cao et al. 2007; Iyer-Pascuzzi et al. 2008). In this study, we attempted to solve the conundrum of the absence of resistance to rice blast despite the presence of the resistance gene *Pb1*in the K209 line. Our studies revealed the exact locus in the gene responsible for rice blast resistance, underscoring the importance of this research in blast resistance breeding. Genetic analysis of KASBL and K209 revealed that the regions Chr8, Chr9, Chr7, and Chr11 were involved in rice blast resistance in at least four rice genomes (Fig. 4). Among them, three QTLs (Chr9, Chr7 and Chr11) negatively affected the resistance despite the presence of *Pb1*. Chr8, on the other hand, had a positive influence on signal transduction, so it is expected that these QTLs will specifically change *Pb1*-mediated resistance single handedly or in combination with others (Fig. 4). This paves the way for creating a line in future containing just the positively acting QTL and excluding the negatively affecting QTLs in order to make a more effective *Pb1*-mediated resistance. Therefore, this result is important in developing highly persistent blast resistance disease varieties by selection of markers.

Investigations into finding the factors responsible for resistance reveal the signal transduction of several factors interacting with each other to produce enhanced resistance to the blast. Although several causative genes have been identified as factors involved in highly quantitative resistance, detailed studies on downstream factors are lacking. Analyses of factors responsible for highly effective, true resistance to rice blast have, however, been progressing. For example, Pit resistance is a true blast resistance gene located downstream of OsRAC1 (Os01g0229400) (Kawasaki et al. 1999; Kawano et al. 2010), OsRAR1 (Os02g0535400) and OsSGT1 (Os01g0624500) (Wang et al. 2008). They are located outside of all the four QTLs loci. Other downstream pathways involved in basic resistance of the blast such as Rice Chitin Receptor CEBiP (Os03g0133400) (Kaku et al. 2006), Rice LysM receptor-like kinase, OsCERK1 (Os08g0538300) (Shimizu et al. 2010), cytoplasmic kinase, OsRLCK176 (Os05g0110900), OsRLCK185 (Os05g0372100), lysin motif-containing proteins, OsLYP6 (Os06g0208800) (Ao et al. 2014) have been shown. They all exist in different QTLs in rice genome. They all exist in different loci in rice genome, except OsLYP4 (Os09g0452200) that may be one of candidates for the QTL on Chr9 (Ao et al. 2014). Altogether, we found that the QTLs of Chr7 and Chr11 are new genes exhibiting resistance to blast disease and this will contribute toward the efforts to elucidate the network of genes responsible for rice blast resistance.

### K209 exhibits lower induction of SA-responsive genes with blast infection compared to KASBL cultivar

When K209 and KASBL cultivars were inoculated with rice blast, we found that *WRKY45 and NH1 were* weakly induced in K209 (Figs. 2b and 3) and the SA concentration was similar to that of KASBL (Fig. 2c). This result indicates that the transport or perception of SA was defective in the K209 cultivar. In *Arabidopsis*, there there is evidence for SA reception by AtNPR proteins (Wu et al. 2012; Fu et al. 2012). Rice has orthologous NPR proteins in the genome, OsNPR1 (Os01g0194300), OsNPR2 (Os01g0767966) and OsNPR3 (Os03g0667100), but these genes did not correspond to the QTLs derived from qualitative reasoning in these experiments.

The mechanism of the induced resistance of *WRKY45* depended on the resistance of *Pb1*, and could not be induced by inoculation with blast disease in this cultivar. The expression of *WRKY45* in rice is induced by BTH by activating the SA pathway (Shimono et al. 2007). Therefore, we investigated whether the induction of *WRKY45* by BTH still remained in these cultivars. In this experiment, the expression of *WRKY45* in both genotypes was maintained normally, and the resistance induced by BTH was conserved (Fig. 2b). However, when treated with rice blast in KASBL and K209 in reproductive growth, the induction of WRKY45 expression was not observed only in the K209 varieties (Fig. 3). Therefore, it is highly probable that the upstream signaling pathway up to WRKY45 is compromised by some mutations in this cultivar for infection of rice blast. Little is known on the upstream signaling activation mechanism of WRKY45 in rice plants. WRKY45 is regulated by several enzymes, including mitogen-activated protein kinase kinase OsMKK10–2 and mitogen-activated protein kinase, some phosphorylation events such as OsMPK4 and OsMPK6 phosphorylation, and also dephosphorylation events such as the tyrosine dephosphorylation of MAPKs by PTPases (OsPTP1/2) in the SA pathway (Ueno et al. 2015). *OsMKK10–2* is located on chromosome 3 (Hamel et al. 2006), and the loci of *OsMPK4* (Chromosome 10), *OsMPK6* (Chromosome 10), *OsPTP1* (Chromosome 12) and *OsPTP2* (Chromosome 11), were all different from the QTLs derived from qualitative reasoning in these experiments. The mechanism of WRKY45 activation against the rice panicle blast of *Pb1*-mediated resistance is still unknown.

## Conclusion

In this report, we have identified the QTL loci of *Pb1* based on the resistance to rice panicle blast with map-based screening. We found that *Pb1* was negatively dependent at least on three QTLs, 7, 9 and 11, and positively on one, QTL 8. This understanding of the molecular mechanism of blast resistance paves the way for creating a line in future containing just the positively acting QTL in order to make a more effective *Pb1*-mediated resistance.

## Methods

### Plant materials

Kanto 209 and Koshihikari Aichi SBL are cultivars that are susceptible and resistant to panicle blast, respectively, despite possessing *Pb1*. 82 F_3_ plants derived from a cross between these cultivars developed by single seed descent method were used for DNA genotyping. The F_4_ lines derived from respective F_3_ plants were used for assessing resistance to panicle blast to detect putative QTLs. To confirm and delimit putative QTLs on chromosome 7, a total of 320 F_4_ or F_5_ lines whose QTL regions were fixed (except for that on chromosome 7) were used to select recombinant inbred plants on and around the putative QTL on chromosome 7. Selected progeny lines (F_5_ or F_6_) were subjected to field tests in 2014, 2015 and 2016.

### DNA sequencing

To sequence *Pb1* and *WRKY45*, we amplified the following regions from the K209 cultivar: for Pb1, we amplified both the Pb1 upstream sequence (−2200 to −1) and ORF region using the primers 5′-AAGGTGAGGTGAGTCATTAGTG-3′ and 5′-TCATGGTTCATTACATTTAA-3′. For WRKY45, we amplified both the upstream sequence (−1700 to −1) and ORF genomic region using the primers 5′-GCCCAATCGGCTGTAATAC-3′ and 5′- AGGCACGTGAAGCTATATGT-3′. The DNA fragments were purified by Nucleo Spin Gel and PCR clean-up kit (Machery-Nagel, Germany). Then, we performed DNA sequencing using BigDye terminator v3.1 Cycle Sequencing kit (ThermoFisher, Japan) with 3130 genetic Analyzer. The obtained sequences were aligned with Nipponbare genome by a plasmid editor software, APE version 2.04 (http://biologylabs.utah.edu/jorgensen/wayned/ape/).

### Evaluation of panicle blast resistance

Panicle blast resistance was evaluated in an experimental paddy field at the Aichi Agricultural Research Center, Mountainous Resin Agricultural Research Institute, where the high pressure of blast disease and its progress are well controlled. Thirteen plants were transplanted for each line in early June. Disease severity was visually evaluated 2–3 weeks after the heading date, and scored from 0 (no diseased grain) to 10 (100% diseased grain) according to the report (Asaga 1981). Some varieties developed for the evaluation of partial resistance to panicle blast (Hayashi et al., 1993) were used for comparison.

### RNA analysis

Total RNA was isolated from rice tissue using Trizol (Invitrogen). For quantitative RT-PCR, total RNA was treated with DNA remover to remove contaminating genomic DNA. cDNA was synthesized using ReverTra Ace reverse transcriptase (Toyobo, Japan). To determine *Pb1* expression, quantitative RT-PCRs were run on a Thermal Cycler Dice TP800 system (Takara Bio, Japan) as shown previously (Hayashi et al. 2010) using primers Pb1sp4Fw/Pb1s4Rv. Rice ubiquitin 1 (Rubq1; AK121590) was used as an internal standard. The primers used in quantitative RT-PCR were listed in Additional file 1: Table S1.

### The measurement of SA and SAG content

Free SA and SAG were quantified as described previously (Raskin et al. 1989; Malamy et al. 1992).

### The SNP genotyping

The genotypes of the F_3_ plants, (which we characterized by using 199 SNP markers), were determined by using 768-plex SNPs for the Illumina Golden Gate Bead Array technology platform (Illumina, Inc., San Diego, CA, USA) based on previously reported information (Ebana et al. 2010; Nagasaki et al. 2010). DNA was extracted from 50 mg of fresh rice leaves using the DNeasy 96 Plant Kit (Qiagen). The designed OPA and 250 ng of DNA were used for the preparation of bead chips according to the protocol for the Golden Gate Genotyping Assay.

### QTL analysis and progeny tests

We constructed a genetic map using MAPMAKER/EXP v. 3.0 software (Lander et al. 1987). QTL analysis for grain length and grain width was performed using version 2.5 of QTL Cartographer software (Basten et al. 2005), and the threshold was obtained by using 1000 permutations. The progeny lines of F_4_ or F_5_ plants were grown to select recombinants within the QTL in order to develop inbred lines with recombinations within and around QTL7. Plants with independent recombination events were selected from on the basis of genotypes around the QTL region determined by using simple-sequence repeat (SSR) markers. The primers for SSR markers were listed in Additional file 1: Table S1.

## Additional file

Additional file 1: Table S1.
*The primers (OsWRKY45, NH1, PR1a, Pb1 and Rubq1*) were used for quantitative RT-PCR analysis. The others (*RM336, RM1017, RM3826, RM5623, RM5847, RM6885, RM21773 and RM21784*) were used for QTL analysis. (PDF 27 kb)
