# Supplementary material for: Panicle blast 1 (Pb1) resistance is dependent on at least four QTLs in the rice genome
Source: Rice (N Y). 2017 Aug 1;10:36. doi: 10.1186/s12284-017-0175-0 (PMC5539066; doi:10.1186/s12284-017-0175-0)
Supplement: Additional file 1: Table S1. — The primers (OsWRKY45, NH1, PR1a, Pb1 and Rubq1) were used for quantitative RT-PCR analysis. The others (RM336, RM1017, RM3826, RM5623, RM5847, RM6885, RM21773 and RM21784) were used for QTL analysis. (PDF 27 kb) [file 12284_2017_175_MOESM1_ESM.pdf]

Table S1. Primers for PCR used in this study

	Left primer	Right primer
OsWRKY45	CGGGTAAAACGATCGAAAGA	TTTCGAAAGCGGAAGAACAG
NH1	GGGAGAAAAGCGGTTCAAAT	TGATGCTTCTCCCGAGTTGTG
PR1a	TCGTATGCTATGCTACGTGTTT	CACTAAGCAAATACGGCTGACA
Pb1	GCAAGACCCAACAGCAAGAC	GATTCGTTCCTCGTCCAATG
Rubq1	GTGGTGGCCAGTAAGTCCTC	GGACACAATGATTAGGGATCA
RM336	GTATCTTACAGAGAAACGGCATCG	GGTTTGTTTCAGGTTCGTCTATCC
RM1017	GATCCGCTTGCACATGGATAGG	TACACGTGTGAGCGAGCTTAGCC
RM3826	TTAGCTTTCCTCCAGTCTCC	ACGGGTATCTGAAACACAAC
RM5623	ATTCCGCCCAGAGGAAGAGTACG	CTGCATGCCACCACACAAACC
RM5847	TGAGATGAGAGATAGACTCC	AACAGATGAAGGCTATTTTA
RM6885	ACACCTTCGGGATCTTACATAGC	CCTCTAGGATACTATGTGGTGACG
RM21773	ACACGACGCAGTTTCCATTCC	GAGACTTTGGATGCTGGCTTGG
RM21784	ACGCAAACGCACGAGAGAGG	CAGCACTGAATCAGGCGAAGC
